# The Teeth and Growth of Children

**Published:** 1901-05-18

**Authors:** 


### THE TEETH AND GROWTH OF CHILDREN.

At the last meeting of the Harveian Society Dr. W. H. Dolamore read a paper in which he pointed out the importance of the first set of teeth, both in regard to health and growth. He first showed how injurious an effect upon the health of the child might be produced by the septic condition of the mouth induced by caries of the deciduous set of teeth. Among other conditions he mentioned ulcerative stomatis, necrosis of bone, and enlarged lymphatic glands, which latter might become the seat of tuberculous growth by which the whole system might become infected. He also traced much gastric disturbance to the same cause. Then he insisted on the important part played by the first teeth in regard to the growth of the child, and he concluded that the mastication of food could not be interfered with by the decay and extraction of deciduous teeth without impairing the health of the child. Discussing this paper Mr. Sefton Sewill thoroughly agreed with the conclusions arrived at by Mr. Dolamore. It was pitiable to see the terrible wreck presented by the mouths of most young children brought to the hospital. But ignorance of the necessity and also of the possibility of preserving the first teeth was not confined to the poorer classes. It was, he said, most important to fill temporary teeth as soon as decay was perceived, rapid onset of root absorption precluding in most cases the possibility of saving them after the pulp had become exposed.

